# Transition between child and adult services for young people with attention-deficit hyperactivity disorder (ADHD): findings from a British national surveillance study

**DOI:** 10.1192/bjp.2019.131

**Published:** 2020-11

**Authors:** Helen Eke, Tamsin Ford, Tamsin Newlove-Delgado, Anna Price, Susan Young, Cornelius Ani, Kapil Sayal, Richard M. Lynn, Moli Paul, Astrid Janssens

**Affiliations:** 1 Postgraduate Research Fellow, University of Exeter Medical School, St. Luke's Campus, UK; 2 Professor of Child and Adolescent Psychiatry, University of Exeter Medical School, St. Luke's Campus, UK; 3 National Institute for Health Research Academic Clinical Lecturer, University of Exeter Medical School, St. Luke's Campus, UK; 4 Associate Research Fellow, University of Exeter Medical School, St. Luke's Campus, UK; 5 Director, Psychology Services Limited, UK; 6 Consultant Child and Adolescent Psychiatrist, Centre for Psychiatry, Imperial College London, UK; 7 Professor of Child and Adolescent Psychiatry, Division of Psychiatry and Applied Psychology, School of Medicine, University of Nottingham; and Professor of Child and Adolescent Psychiatry, CANDAL (Centre for ADHD and Neuro-Developmental Disorders across the Lifespan), Institute of Mental Health, UK; 8 British Paediatric Surveillance Unit, Royal College of Paediatrics and Child Health, UK; 9 Consultant Child and Adolescent Psychiatrist, Coventry and Warwickshire Partnership Trust, UK; 10 Associate Professor, Department of Public Health, University of Southern Denmark, Denmark; and Honorary Associate Professor, University of Exeter Medical School, St. Luke's Campus, UK

**Keywords:** Attention-deficit hyperactivity disorder, surveillance, British Paediatric Surveillance Unit (BPSU), Child and Adolescent Psychiatry Surveillance System (CAPSS), incidence

## Abstract

**Background:**

Optimal transition from child to adult services involves continuity, joint care, planning meetings and information transfer; commissioners and service providers therefore need data on how many people require that service. Although attention-deficit hyperactivity disorder (ADHD) frequently persists into adulthood, evidence is limited on these transitions.

**Aims:**

To estimate the national incidence of young people taking medication for ADHD that require and complete transition, and to describe the proportion that experienced optimal transition.

**Method:**

Surveillance over 12 months using the British Paediatric Surveillance Unit and Child and Adolescent Psychiatry Surveillance System, including baseline notification and follow-up questionnaires.

**Results:**

Questionnaire response was 79% at baseline and 82% at follow-up. For those aged 17–19, incident rate (range adjusted for non-response) of transition need was 202–511 per 100 000 people aged 17–19 per year, with successful transition of 38–96 per 100 000 people aged 17–19 per year. Eligible young people with ADHD were mostly male (77%) with a comorbid condition (62%). Half were referred to specialist adult ADHD and 25% to general adult mental health services; 64% had referral accepted but only 22% attended a first appointment. Only 6% met optimal transition criteria.

**Conclusions:**

As inclusion criteria required participants to be on medication, these estimates represent the lower limit of the transition need. Two critical points were apparent: referral acceptance and first appointment attendance. The low rate of successful transition and limited guideline adherence indicates significant need for commissioners and service providers to improve service transition experiences.

To plan services, commissioners and service providers need accurate and timely data on how many people may require that service. There are currently limited national and international data on the number of young people with attention-deficit hyperactivity disorder (ADHD) who need transition to adult services when they become too old for child services. ADHD affects approximately 5% of the childhood population, 15% of which still meet the full, formal diagnostic criteria at age 25 and up to 65% still have impairing symptoms at age 25 but may not meet the full formal criteria.^1,2^ Consequently, there is a group of young people in need of continued support for the management of ADHD in early adulthood. There are few empirical data on the number of young people with ADHD who wish to access ongoing care in adulthood, or the number that successfully do so. Some studies have attempted to quantify national estimates for transition, but they have either focused on all neurodevelopmental conditions rather than just ADHD or were limited geographically.^3,4^

The current study aimed to estimate the incidence of young people with ADHD who need transition from child and adolescent services to adult services across the UK and Republic of Ireland (henceforth, the British Isles). For the purposes of this study, transition refers to the transfer of care from child and adolescent mental health services (CAMHS) or paediatric services to an adult service for continued treatment for ADHD once the young person reaches the service transition boundary. The main objectives of the study were to describe the group of young people diagnosed with ADHD who require medication beyond the end of children's services in terms of range and mean age for transition, to estimate the incidence rate of young people with ADHD who require ongoing medication for ADHD after they pass the age boundary for the child service, to estimate the proportion of young people with ADHD judged in need of transition to adult mental health services due to ongoing need for medication that successfully transferred to a specialist health service, and to describe the proportion of young people who experience optimal transition. Successful transition was defined as a referral to an adult service made, accepted and the first appointment attended; and optimal transition as joint care, planning meetings, information transfer and continuity of care.^5^ These definitions are in line with recommendations in the current National Institute for Health and Care Excellence (NICE) guidelines for ADHD.^6^

## Methods

This study, which was part of a research programme that explored transition in ADHD (CATCh-uS) funded by the National Institute for Health Research (HS&DR 14/21/52),^7^ used the British Paediatric Surveillance Unit (BPSU) and the Child and Adolescent Psychiatry Surveillance System (CAPSS) to collect prospective data on the number of young people with ADHD who undergo transition from children's services to adult mental health services. These surveillance units provide a method that allows the collection of reliable national estimates of service level incidence about rare health conditions or events in paediatrics and child mental health services to improve their identification and clinical management. The surveillance methodology is described in more detail elsewhere but is briefly summarised below.^8–10^

Young people taking medication for a clinical diagnosis of ADHD who were requiring transition to an adult service for continued treatment were notified prospectively using the BPSU and CAPSS methodology over 13 months from 1 November 2015 to 30 November 2016. The first (pilot) month was discarded as per BPSU and CAPSS protocol. Consultant paediatricians and consultant child and adolescent psychiatrists in the British Isles were systematically prompted by a monthly email or postal reporting card which asked them to indicate the number of eligible young people with ADHD they had seen in the previous month or ‘nothing to report’. Details regarding each reported case were subsequently gathered by study investigators using a notification questionnaire sent to the reporting clinician. Information on the outcome of the transition of eligible young people was collected using a follow-up questionnaire 9 months after notification. Baseline notification and follow-up questionnaires were developed using the BPSU and CAPSS templates, which comprised structured questions (30 at baseline and 19 at follow-up) with two open text responses. A copy of the questionnaires used can be found as Supplementary Material available at https://doi.org/10.1192/bjp.2019.131. A follow-up questionnaire regarding those individuals confirmed as eligible at baseline was sent to paediatricians and psychiatrists 9 months later. Duplicate reporting of cases was checked by matching minimal identifiers.

The study was approved by both BPSU and CAPSS Executive Committees. Health Research Authority and Confidentiality Advisory Group (CAG) approvals permitted access to case note information without patient/parent consent (Integrated Research Application System registration number 159209, REC reference 15/YH/0426, CAG reference 15/CAG/0184).

### Case definition criteria for notification

The case definition criteria were developed to be itemised and precise and to specify the need for the young person to have ongoing support for medical treatment from specialist adult mental health services, as outlined in the NICE guidelines.^6^ It was designed in close collaboration with both BPSU and CAPSS to ensure that both paediatricians and child and adolescent psychiatrists would identify young people in a similar manner. The surveillance asked for young people seen in the previous month to be reported if they were judged to meet the following criteria by the reporting clinician.

(a) Clinical diagnosis of ADHD, under the care of CAMHS or paediatrics, reviewed within 6 months of the service's upper age (transition) boundary.
(b) Considered to require and willing to take continued medical treatment for symptoms of ADHD after crossing the transition boundary of the child service.
(c) Comorbid diagnoses, including intellectual/developmental disabilities, were included *only* if it was the ADHD that required ongoing medical treatment in adulthood.

### Data analysis

Analysis of data was descriptive. Response rates at each stage of the study are described, as are sociodemographic details of the reported individuals. An incidence rate is defined as the number of new health-related events, in a defined population, during a stated period of time.^11^ The incidence rate of transition was calculated by dividing the number of confirmed young people with ADHD who need transition identified over the course of the study's 12 month surveillance period by the population at risk. The population at risk was derived by applying the estimated prevalence of ADHD (approximately 5% in the child and adolescent population)^1^ to the total number of children aged 17–19 years in the British Isles as reported in 2016 (*n* = 2 333 035).^12^ The quotient was then multiplied by 100 000 to provide the incidence rate of transition per 100 000 population of people aged 17–19 per year. Two incidence rates were calculated: the incidence of young people who required transition as defined by the case definition criteria and the incidence rate of successful transition in the obtained sample, defined as those whose referrals were accepted and attended their first appointment in the adult service. The observed incidence rate was adjusted to take into account the age of the young person, the current NICE guidance about the age of transition (18 years) and missing data (failures to notify or return questionnaires) as suggested in a previous study (see Table 2).^13^

### Data availability

Data are currently stored securely by the University of Exeter Medical School. Data are under embargo until the end of the CATCh-uS project (2019).

## Results

Table 1 illustrates the return of questionnaires for each stage of the surveillance study. The mean monthly response rate was 94% in BPSU and 53% in CAPSS. A total of 614 eligible young people were reported by 249 different clinicians. The overall response rate to the baseline questionnaire was 90% from BPSU and 67% from CAPSS clinicians, and at follow-up it was 84% and 80%, respectively. The response rates include contacts with clinicians who provided an explanation for not returning the questionnaire, including for reasons such as inability to recall the patient reported, reporting the individual in error or subsequent realisation that the individual did not meet the definition criteria.

**Table 1:** Surveillance study data November 2015–November 2016

Baseline (% based on total reported cases)	BPSU (*n* = 314)	CAPSS (*n* = 300)	Combined (*n* = 614)
Not returned – received clear explanation for why	29 (9%)	27 (9%)	56 (9%)
Not returned – no explanation	41 (13%)	127 (42%)	168 (27%)
Duplicate cases	6 (2%)	7 (2%)	13 (2%)
Returned baseline questionnaire	238 (76%)	139 (46%)	377 (61%)
Ineligible cases	36 (11%)	26 (9%)	62 (10%)
Eligible cases	202 (64%)	113 (38%)	315 (51%)
Follow-up (% based on total eligible cases)	BPSU (*n* = 202)	CAPSS (*n* = 113)	Combined (*n* = 315)
Returned follow-up questionnaire	161 (80%)	86 (76%)	247 (78%)
Not returned – received clear explanation for why	12 (6%)	8 (7%)	20 (6%)
Not returned – no explanation	29 (14%)	19 (17%)	48 (15%)

BPSU, British Paediatric Surveillance Unit; CAPSS, Child and Adolescent Psychiatry Surveillance System.

No individuals were reported through both BPSU and CAPSS. A total of 13 duplicate reports were identified from clinicians who reported the same individual more than once during the surveillance period. A total of 17 questionnaires could not be completed at follow-up as the clinician no longer had access to the patient's records or was no longer in post. Some questionnaires at baseline and follow-up were returned blank or not fully completed (*n* = 86). However, information from partially completed questionnaires was included in the analysis. The 315 eligible individuals were reported by 148 different clinicians.

### Demographics of young people reported

The population of young people reported was largely male (77%) and White British (91%). Individuals were reported from across the British Isles but most (over 85%) were seen in England. The modal age boundary between child and adult services was 18 years old, but ranged from 14 to 19 years. Two individuals who did not originate from the British Isles were international students seen in private practice in England. The reported age range of reported individuals extended from 14 to 20 years, although 85% were aged 17 to 19 years at the point of referral for transition, and age was not reported for 6% of individuals.

A large proportion of individuals (56% from paediatricians, 68% from psychiatrists) were reported to have a comorbid condition; for 25% the comorbidity was an autism spectrum disorder. Polypharmacy was common: 23% of patients from paediatricians and 41% from psychiatrists were prescribed more than one medication.

### Incidence of transition

Table 2 demonstrates the incidence calculations, adjusted for age and non-response. In total, there were 315 confirmed eligible cases (202 BPSU, 113 CAPSS). Follow-up questionnaires were received for 247 cases; 55 of these (22 BPSU, 33 CAPSS) confirmed that a successful transition was achieved (i.e. a referral made, accepted and the young person attended first appointment in the adult service). When only the individuals aged 17 to 19 years were extracted from these data, there remained 269 eligible for transition and 51 that were reported to have had a successful transition.

**Table 2:** Calculation of observed and adjusted incidence rate of successful transition for individuals aged 17–19 years (per 100 000 people aged 17–19 per year)

	Incidence rate
Observed incidence:	
Incidence: eligible for transition (all eligible individuals identified in 12 months) per 100 000 per year	(315/116 651) × 100 000 = 270.0
Incidence: successful transition (referral made, accepted and first appointment attended) per 100 000 per year	(55/116 651) × 100 000 = 47.1
Incidence: eligible for transition aged 17–19 (all eligible individuals aged 17–19 identified in 12 months) per 100 000 per year	(269/116 651) × 100 000 = 230.6
Incidence: successful transition aged 17–19 (referral made, accepted and first appointment attended) per 100 000 per year	(51/116 651) × 100 000 = 43.7
Correction for non-returned notification cards (no age known):	
Returned	73.7%
No response	26.3%
Assumption 1: observed incidence applies to half (13.2) of non-returned (26.3%) cards because clinicians are more likely to respond with cases to report	(13.2 + 26.3)/73.7 = coefficient 0.54
Assumption 2: observed incidence applies to all non-returned cards; assumes no difference in incidence between cases that were reported and not reported	100/73.7 = 1.36
Correction for non-returned baseline questionnaires (no age known):	
Returned	377/614 = 61.4% 100/61.4 = coefficient 1.63
Combined coefficients for individuals aged 17–19 only:	
Adjusted incidence rate 1 = incidence rate × correction for unreturned notification cards (assumption 1) × correction for unreturned baseline questionnaires	Eligible for transition: 230.6 × 0.54 × 1.63 = **202.9** Successful transition: 43.7 × 0.54 × 1.63 = **38.5**
Adjusted incidence rate 2 = incidence rate × correction for unreturned notification cards (assumption 2) × correction for unreturned baseline questionnaires	Eligible for transition: 230.6. × 1.36 × 1.63 = **511.2** Successful transition: 43.7 × 1.36 × 1.63 = **96.9**

Figures in bold estimate the range for eligible and successful transition.

Figures in bold in Table 2 estimate the range for eligible and successful transition. The adjusted incidence rates provide a likely range within which the actual rate is likely to fall, and suggest between 202.9 and 511.2 per 100 000 17–19 year olds per year were eligible, but successful transition was less common (38.5 and 96.9 per 100 000 young people aged 17–19 years per year).

### Transition quality and outcomes

Half of all the reported individuals (regardless of age at referral) were referred to a specialist adult ADHD service, just over a quarter to general adult mental health services and 10% were referred back to primary care. Referral destinations were similar regardless of whether the young person was reported by a paediatrician or a psychiatrist.

**Fig. 1:**
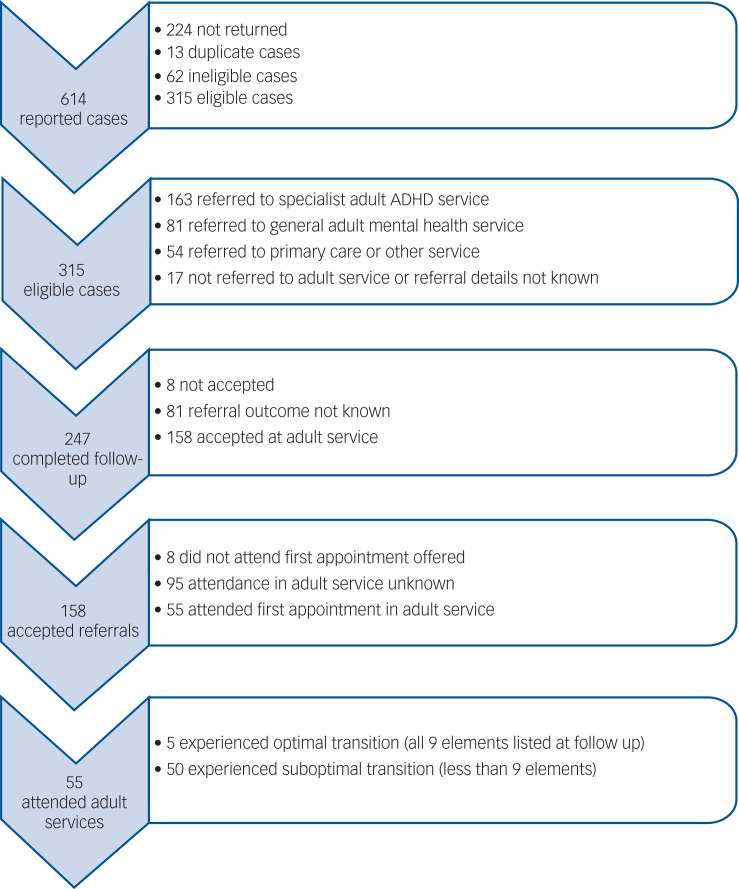
Reported cases, referral details and optimal transition outcome. ADHD, attention-deficit hyperactivity disorder.

In total, 64% (*n* = 158) of the 247 individuals who were referred to adult mental health services were accepted (BPSU 52%, CAPSS 86%), but only 22% (*n* = 55) were reported to have attended a first appointment (14% BPSU, 38% CAPSS) (Fig. 1). Reported reasons for failed transitions included: the patient disengaged and no longer wanted to take medication (*n* = 3), the referral did not meet adult service criteria (*n* = 1), there was no funding available (*n* = 1) or the adult service was closed to new referrals due to lack of resources or long waiting lists (*n* = 4). No reason for a failed transition was given for the remaining individuals (*n* = 46).

Nearly all (93%) clinicians reported that the young person had been involved in the planning of the transition process, and over 80% reported that the parent or carer was also involved. More child and adolescent psychiatrists than paediatricians reported access to (81% *v.* 39%) and use of (66% *v.* 36%) a transition protocol in their organisation.

At baseline notification only 6% of paediatricians and 10% of psychiatrists indicated that all five optimal criteria pre-transition (see Table 3) were apparent in the transition planning. At follow-up only 2% of paediatricians and 6% of psychiatrists considered that there was adherence to all nine optimal criteria post-transition. Some elements were reported considerably less frequently at follow-up than at baseline, which suggests that clinicians anticipate being able to complete these elements, but when providing a retrospective report at follow-up some elements may either have not been recalled or were not carried out. These included: information sharing (84.6% at baseline *v.* 68.8% at follow-up), young person involvement (81.4% *v.* 69.6%) and joint working (25.5% *v.* 10.5%).

**Table 3:** Factors of optimal transition reported pre- and post-transition

Pre-transition	BPSU (*n* = 202)		CAPSS (*n* = 113)		Combined (*n* = 315)	
	Total ‘yes’ response	%	Total ‘yes’ response	%	Total ‘yes’ response	%
Information sharing	176	87.1	93	82.3	269	84.6
Young person involvement	162	80.2	97	85.8	259	81.4
Planning meeting	23	11.4	29	25.7	52	16.3
Plan and agree care plan	49	24.3	46	40.7	95	29.9
Handover period	56	27.7	25	22.1	81	25.5

Post-transition	BPSU (*n* = 161)		CAPSS (*n* = 86)		Combined (*n* = 247)	
	Total ‘yes’ response	%	Total ‘yes’ response	%	Total ‘yes’ response	%
Patient/carer involvement	116	72	56	65.1	172	69.6
Information sharing	105	65.2	65	75.6	170	68.8
Care plan agreed	35	21.7	44	51.2	79	32.0
Joint working before transfer	12	7.5	14	16.3	26	10.5
Alignment of assessment procedures	9	5.6	12	14.1	21	8.5
Continuity of care	35	21.7	41	47.7	76	30.8
Consistency of care	13	8.1	36	41.9	49	19.8
Consideration of appropriate service	78	48.4	50	58.1	128	51.8
Clarity of funding and eligibility	66	41.1	51	59.3	117	47.4

BPSU, British Paediatric Surveillance Unit; CAPSS, Child and Adolescent Psychiatry Surveillance System.

## Discussion

This surveillance study generated the first national data to estimate the number of young people with ADHD taking medication who require and complete a transition to an adult service in the British Isles. Our findings suggest that the annual scale of the need for young adults with ADHD who require transition to adult services for ongoing medication in the British Isles lies between 202.9 and 511.2 per 100,000 17–19 year olds per year. Given the study's inclusion criterion that the eligible young person had to need and want to continue with medication for ADHD after reaching the age boundary of the children's service, which does not take into account the demand for psychological support, these figures are likely to be a considerable underestimate of the actual need for service provision. Further, a comparison of the surveillance data collected in the current study with the Clinical Records Interactive Search (CRIS) at the South London and Maudsley National Health Service (NHS) Trust (SLaM) highlighted that surveillance with CAPSS only identified 25% of potential ADHD transition cases in the London area. Details regarding this study are available from the author on request. Sadly, there were no comparable data to triangulate with BPSU reports, but the CRIS data emphasise that although our estimates should therefore be treated as extremely conservative, they remain the best empirically based British Isles data available for service commissioners and providers. The use of population data restricted to age 17–19 is a limitation of the study and may have therefore excluded relevant cases, however it reflects 85% of eligible reported cases and the NICE guidance that transition should occur by age 18. The requirement that reported individuals needed ongoing medication aimed to increase reliability by having an unequivocal reference for the reporting clinician.

Previous studies have only been able to estimate the number of transition cases in smaller localities that are difficult to compare directly with our findings. A London-based study suggested an average of 12 neurodevelopmental individuals annually per CAMHS team that require a transition to an adult service, with 8 of the 12 making the transition successfully.^3^ A study from the Republic of Ireland used the same methodology and found 20 ADHD cases from 4 CAMHS teams annually requiring transition, with only 3 individuals successfully transitioning to an adult service.^4^ Similarly there are a lack of comparative international data on transition in ADHD; an ongoing European study on transition focuses on mental health transition generally but is not specific to ADHD.^14^ Given the rise in prescriptions for ADHD over the past decades,^15^ estimates may quickly become out of date as later cohorts are likely to contain a higher proportion of young adults who may have benefitted from medication and therefore wish to continue to take it. A recent report reviewing children and young people's mental healthcare highlighted a lack of data availability and monitoring of transition^16^ and reviews such as this only consider young people up to the age of 18, so knowledge of the needs of young adults in their later teens or early twenties is poor.

The estimated annual incidence of successful transitions lies between 38.5 and 96.9 per 100 000 young people aged 17–19 years per year, which suggests that only a fifth of those requiring transition for ongoing medication successfully made the transfer. A small proportion of failed transitions related to the young person disengaging from services or their medication, which would render them ineligible by our definition, but it may also relate to the lack of suitable services for onward referral. A study of a locality in North West England reported that only 15% of patients eligible for transition actually successfully transferred to the adult service.^17^ Others have demonstrated above predicted levels of medication cessation between the ages of 15 to 18, before transition, which may be influenced by the lack of availability of services.^18^ Data from UK primary care suggested that only 18% were still taking medication for ADHD at age 18.^15^ These findings suggest a worrying discontinuity of service between child and adult services, given that patient registry studies of young adults who discontinue their medication show poorer outcomes compared with those who continue to take it.^19^ Given the number of young people reported in this surveillance study that did not attend the first appointment in the adult service, it is possible that the transition referral for ongoing treatment might reflect a clinician decision regarding the need for treatment, rather than a decision made by the young person.

Our findings suggest poor adherence to the recommendations for transition from the NICE guidelines for ADHD. NICE recommend that a good transition between child and adult services should be complete by age 18, involve a detailed care plan, include a formal joint meeting between the child and adult service, use the care-programme approach and involve the young person and the parent or carer.^6^ The guidelines do not specify what type of adult service a young person should be transitioned to, they only state adult mental health services, and encouragingly over 75% of individuals in this study were referred to either a specialist ADHD or adult mental health service. In contrast, we found that a joint planning meeting, a care plan and a joint handover period were conducted in fewer than 30% of cases. Other studies have also highlighted the lack of planning for transition of young people with ADHD.^3,4,20,21^ Although the reported high level of involvement of the young person and carer in the process is commendable, paediatricians in particular reported poor continuity and consistency of care. This may reflect weaker links between paediatricians and adult mental health services when compared with CAMHS. A lack of planning is likely to undermine the potential for successful transition, and the need to adhere to recommendations to ensure effective transition has been highlighted.^22^ Further, it is recommended that policies and guidelines are reviewed regularly so they can be operationalised and effectively translated into clinical practice.^23^ A systematic review of guidelines has suggested that this does not occur; guidelines are often not incorporated into protocols locally and do not always reflect the clinical reality.^24^

The use of the BPSU and CAPSS systems provided national-level, prospectively collected data but also presented a number of methodological challenges. Registration to receive the monthly reporting cards is voluntary and mostly consists of those in the consultant grade. Therefore not all relevant clinicians may receive them (although doctors from non-consultant grades and non-medical staff may report cases via the consultant). This is likely to be the main explanation for the discrepancy between CAPSS and the CRIS case note review. Details of this study are available from the author on request. Other research has demonstrated that patients may be reviewed in settings other than paediatrics and CAMHS, such as primary care or forensic services,^25^ who would not ordinarily be reached by either surveillance system. There is also a relative underrepresentation of clinicians reporting to the surveillance units in the private sector despite its increasingly important role in healthcare provision,^26^ which may particularly be an issue for young adults with ADHD for whom there are few NHS services.^7^ Indeed, our findings highlighted referral back to primary care in 10% of cases. Incomplete data also presented a limitation: some contact details provided by both surveillance organisations were not up to date and some questionnaires were returned blank or with missing data.

Although the return rate of reporting cards by paediatricians via BPSU was excellent, perhaps due to longevity of the system,^27^ the average return rate was much lower in CAPSS. CAPSS was set up more recently (2009), so is perhaps less ingrained in clinical practice for child and adolescent psychiatrists than BPSU is for paediatricians. The lower return rate may reflect a lack of awareness of the system and not necessarily a reflection of clinicians actively being non-compliant. Potential difficulties with the case definition could also have led to a lack of reported cases. Previous surveillance studies have also cited difficulties with reporting, case definitions and low return rates.^28–31^ Research is enshrined in the NHS constitution as a core activity,^32^ however clinicians reported that current workloads made it difficult to respond to questionnaires and some service providers did not support their clinicians to participate. We provided certificates for questionnaire completion that indicated involvement in research for appraisals, but it is clear that busy clinicians need more support and encouragement to engage with research.

Surveillance methodology has stringent governance and requires considerable researcher time for data collection and analysis, but has offered a more complete national picture of the need and success of transition to adult service among young people with ADHD than previous studies have achieved. Surveillance allows researchers to ask a wider and more tailored set of questions than case note review alone. The findings emphasise a relative lack of adherence to recommended guidelines for transition and the low proportion of eligible patients that experience successful transition and a continuity of care.

Attempts have been made to correct for incomplete ascertainment and to provide a series of transparent estimates for policy, commissioning and service provision. Despite some limitations, to our knowledge these data are the best currently available. European studies have similarly highlighted a lack of transition policy^14^ and the societal impact of ADHD if not managed.^19^ Our findings are significant for commissioners and service providers, internationally as well as in the British Isles, to address the drop in attendance from child to adult services. It is imperative that mental health services develop policy and strategy to better support this group of young people in the future.

## Supplementary material

For supplementary material accompanying this paper visit https://doi.org/10.1192/bjp.2019.131. click here to view supplementary material
