# Supplementary material for: Transition between child and adult services for young people with attention-deficit hyperactivity disorder (ADHD): findings from a British national surveillance study
Source: Br J Psychiatry. 2020 Nov;217(5):616–22. doi: 10.1192/bjp.2019.131 (PMC7589988; doi:10.1192/bjp.2019.131)
Supplement: Supplementary file 1 [file S0007125019001314sup.zip › S0007125019001314sup001.pdf]

Case notification form Questionnaire – Strictly Confidential

CATCh-uS (Children with ADHD in transition between children's and adult services)

The first page of the case notification form will be stored separately from the rest of the questionnaire and personal identifying information for the case (young person) will be used only for linkage of records.

Reporting Instructions:

Please report any young person with ADHD taking medication for ADHD seen by you for the first time in the six months preceding the young person reaching your service's age boundary. Please report any case even if you believe the case may have been reported from elsewhere.

Case Definition:

Section A: Reporter Details

1.1 Date of completion of questionnaire: D D / M M / Y Y Y Y

1.2 Consultant or specialist responsible for case: ______________________

1.3 Name of clinic and Trust/Provider: ______________________

1.4 Telephone number: ______________ Email: ______________

Section B: Case Details

2.1 NHS/CHI No: ☐☐☐ ☐☐☐ ☐☐☐☐

2.2 Hospital No: ☐☐☐☐☐☐☐☐☐☐

2.3 First half of postcode only ☐☐☐☐ Town of Birth (if ROI) _ _ _ _ _ _ _ _

2.4 Sex: M ☐ F ☐ Age of case (Years/months) _ _ _ _ _ _ _ _

2.5 Ethnicity*: ☐☐ Specify if any "Other" background: _ _ _

**Please choose the correct ethnicity code from Appendix A overleaf*

Appendix A

Appendix A: Coding for Ethnic Group (ONS 2011 for UK wide data collection)

		Ethnicity Code
**A**	**White**	
	English / Welsh / Scottish / Northern Irish / British	**1**
	Irish	**2**
	Gypsy or Irish Traveller	**3**
	Any other White background, please describe	**4**
**B**	**Mixed/ Multiple Ethnic Groups**	
	White and Black Caribbean	**5**
	White and Black African	**6**
	White and Asian	**7**
	Any other Mixed / Multiple ethnic background, please describe	**8**
**C**	**Asian / Asian British**	
	Indian	**9**
	Pakistani	**10**
	Bangladeshi	**11**
	Chinese	**12**
	Any other Asian background, please describe	**13**

		Ethnicity Code
**D**	**Black / African / Caribbean / Black British**	
	African	**14**
	Caribbean	**15**
	Any other Black / African / Caribbean background, please describe	**16**
**E**	**Other ethnic group**	
	Arab	**17**
	Any other ethnic group, please describe	**18**

Section C: Eligibility of case

3.1 Does the young person meet the following criteria for this study?

	Yes	No
1. Does the young person have a clinical diagnosis of ADHD?	☐	☐
2. Is the young person currently receiving drug treatment for their ADHD?	☐	☐
3. Does this case require continuation of their drug treatment for their ADHD after transition from your service (i.e. in adult services)? *Note:* please ONLY tick 'yes' if this drug treatment is required for their ADHD rather than any existing comorbid diagnosis.	☐	☐
4. Is this case within six months of the age boundary for your service? – i.e. in ideal circumstances, within six months of transition?	☐	☐
5. Is this the first time this case is being reported to this study by your service? *Note:* Please only report a case once - those who have already been seen and reported by you in this time-scale should not be reported a second time.	☐	☐
**3.2 Does this case meet all of the five criteria (yes to all questions)**	☐	☐

- **If so, please continue with the questionnaire.**
- **If not, thank you again for your time. There are no further questions to answer; please proceed on page 6 of this questionnaire.**

Section D: Comorbidities and medication

4.1 Aside from their clinical diagnosis of ADHD, does this case have any other mental health or developmental diagnoses?

☐ Yes ☐ No ☐ Not known to me

Please list any other diagnoses below:

☐ Autism spectrum condition
☐ Chronic Tic disorder / Tourette's
☐ ODD / Conduct disorder
☐ Anxiety disorder
☐ Dyspraxia
☐ Problematic substance abuse
☐ Other? Please specify: . . . .
. . . .

4.2 Please list below the medication which the young person is currently prescribed for any mental health / developmental conditions and the indication. Please also indicate whether you consider that this medication requires continuation beyond the age boundary for your service.

Medication and indication	Requires continuation: Yes	No	Don't know
______	☐	☐	☐
______	☐	☐	☐
______	☐	☐	☐

Section E: Referral of the case

5.1 What is the age boundary for your service?

_ _ years _ _ months

5.2 What is the current status of this case regarding reaching the age boundary of your service?

I last saw the young person _ _/ _ _/ _ _ _ _

Has the young person already reached the age boundary for your service? ☐ Yes ☐ No

Do you have another appointment with the young person? ☐ Yes ☐ No

Are you still responsible for the young person? ☐ Yes ☐ No

5.3 Have you started the transition process yet?

☐ Yes – Please continue this questionnaire.

☐ No – Please go to page 6 of the questionnaire.

5.4 What is the intended destination for this young person following transfer from your service, for the management of their ADHD? Please provide name and or contact details of the service.

☐ Specialist Adult ADHD service: _

☐ Other Adult Mental Health Service: _

☐ Primary care / GP: _

☐ No specific arrangements are made

☐ Other. Please give details or any other comments below:

Section F: The transition protocols and procedures

6.1 Does your organisation have a transition protocol?

☐ Yes ☐ No

6.2 Are you using it to plan the transition for this case?

☐ Yes ☐ No

Section G: Facts regarding the transition of the case

7.1 Key stages in the transition process: which of the following steps have you undertaken? Please give an indication of time if you have engaged in this element of the transition process (DD/MM/YYYY).

When did you first discuss a transfer to an adult service with your case?

Date: _ _ _ _ _ _ _ _ _ _ _ _ _ _ _ _ ☐ Not yet ☐ Not known to me

When did you first refer the young person to an adult service?

Date: _ _ _ _ _ _ _ _ _ _ _ _ _ _ _ _ ☐ Not yet ☐ Not known to me

How many services did you approach to find a match for your case?

☐ Just one ☐ More than one: _ _ _ _ _

If a referral was made, was the referral accepted?

☐ Yes, Date: _ _ _ _ _ _ _ _ _ _ _ _ ☐ No ☐ I am awaiting a response

7.2 Partners involved: State which of the following partners are involved in the transition process:

	Yes	No	Not known
Young person	☐	☐	☐
Parents	☐	☐	☐
GP	☐	☐	☐
Care co-ordinator from adult team	☐	☐	☐
Care co-ordinator from child team	☐	☐	☐

Other? Please specify: _

7.3 Which of the following elements of the transition process have been initiated:

	Yes	No	Not known
Information sharing between services (case notes or summaries)	☐	☐	☐
Young person's involvement in decision making	☐	☐	☐
Organising a transition planning meeting (involving the young person and carer, and key professionals of both services)	☐	☐	☐
Planning and agreeing on a care plan	☐	☐	☐
A period of handover or parallel/joint care	☐	☐	☐

Other elements you want to add:

___

___

___

Section H: Request to take part in follow-up

8.1 We wish to interview a sample of clinicians about their general experiences of managing transition, using a semi-structured telephone interview that will take approximately 30 minutes. Would you be willing to be contacted regarding taking part in such an interview? (This does not constitute any obligation to take part). We will not be discussing individual cases.

☐ Yes ☐ No

Thank you for taking the time to complete the questionnaire

Please print and return the completed form in the SAE.

If you have any questions about the study please do not hesitate to contact the investigators by email or telephone :

Telephone: ______________________________ Email: ______________________________

Ethical approval

This study has been approved by NRES South Yorkshire Ethics Committee – Yorkshire & The Humber (REC Reference: 15/YH/0426) and has been granted **Section 251 HRA-CAG permission (CAG Reference: 15/CAG/0184)**.
