# Supplementary material for: Transition between child and adult services for young people with attention-deficit hyperactivity disorder (ADHD): findings from a British national surveillance study
Source: Br J Psychiatry. 2020 Nov;217(5):616–22. doi: 10.1192/bjp.2019.131 (PMC7589988; doi:10.1192/bjp.2019.131)
Supplement: Supplementary file 1 [file S0007125019001314sup.zip › S0007125019001314sup002.pdf]

Follow-up Questionnaire – Strictly Confidential

CATCh-uS (Children with ADHD in transition between children's and adult services)

The first page of the case notification form will be stored separately from the rest of the questionnaire and personal identifying information for the case will be used only for linkage of records.

Reporting Instructions:

This questionnaire has been sent to you as you have identified a case 9 months ago. You reported a young person with ADHD taking medication for their ADHD seen by you six months before the young person reached your service's age boundary. Please could you answer the following question regarding this patient?

Ethical approval:

This study has been approved by NRES South Yorkshire Ethics Committee – Yorkshire & The Humber (REC Reference: 15/YH/0426) and has been granted Section 251 HRA-CAG permission (CAG Reference: 15/CAG/0184).

Case Definition:

Section A: Reporter Details

1.1 Date of completion of questionnaire: D D / M M / Y Y Y Y

1.2 Consultant responsible for case: ______________________

1.3 Name of clinic and Trust/Provider: ______________________

1.4 Telephone number: ____________ Email: ____________

Section B: Case Details

2.1 NHS/CHI No: ☐☐☐ ☐☐☐ ☐☐☐☐

2.2 Hospital No: ☐☐☐☐☐☐☐☐☐☐

***Note*: Abbreviation used: YP – Young person**

Thank you for taking the time to complete the questionnaire

Please print and return the completed form in the SAE.

If you have any questions about the study please do not hesitate to contact the Principal Investigator by email or telephone :

Telephone: Email:

Section C: Eligibility of case

3.1 ADHD medication

At the time of transfer was the YP prescribed medication for their ADHD?

☐ Yes
- ☐ Methylphenidate
- ☐ Atomoxetine
- ☐ Lisdexamphetamine
- ☐ Dexamfetamine
- ☐ Clonidine
- ☐ Atypical Antipsychotics
- ☐ Other, Please specify: . . . .

☐ No

Section D: Destination of case

4.1 When did you last see the young person?

Date: _ _ / _ _ / _ _ _ _

4.2 Where was the young person referred to for the management of their ADHD? Please give name and contact details (telephone or email).

☐ Specialist Adult ADHD service: ………………………………………………

☐ Other Adult Mental Health Service: ………………………………………………..

☐ Primary care / GP: ……………………………………………………………

☐ No specific arrangements were made

☐ Other: please state here: ……………………..……………………………………
……………………………………………………………………………………………

4.3 Has the referral been accepted?

☐ Yes Date: _ _ / _ _ / _ _ _ _

☐ No Please go to section F.

☐ I don't know

Section E: Facts regarding the transition of the case

5.1 Have you received any feedback from the service to which you referred the YP?

☐ Yes - Please continue with the questionnaire ☐ No – Skip Q. 5.2

5.2 Response from the service to which you referred the YP:

	Yes	No	I don't know
Did the young person receive an appointment?	☐	☐	☐
Did the young person attend the appointment?	☐	☐	☐

Section F*: Facts regarding a refused referral

*** Only for cases that did not get accepted at the service to which you referred the YP.**

6.1 **Have you received any feedback from the service to which you referred the YP as to why the YP was not accepted? If so, please specify.**

☐ Yes: ……………………………………………………………………….. ☐ No

………………………………………………………………………………………

6.2 **Do you know whether the young person currently receives care for their ADHD elsewhere? If so, could you please give the name of the service/treating clinician?**

☐ Yes: ……………………………………………………….. ☐ No ☐ I don't know

…………………………………………………………………………

Section G: Elements of optimal transition

7.1 **In your opinion/experience, were the following elements or processes present in the transition of this young person from your service?**

	Yes	To some extent	No	Not known
User/carer involvement in decision	☐	☐	☐	☐
Information sharing between services	☐	☐	☐	☐
Has a care plan been agreed?	☐	☐	☐	☐
Joint working preceding transfer	☐	☐	☐	☐
Alignment of assessment procedures between services	☐	☐	☐	☐
Continuity of care	☐	☐	☐	☐
Consistency of care	☐	☐	☐	☐
Consideration of most appropriate service	☐	☐	☐	☐
Clarity of funding arrangements and/or eligibility for adult services	☐	☐	☐	☐

7.2 **Please add any other comments you have regarding whether transition was optimal in this case:**
